# Psychological Adjustment in Patients with Anorexia Nervosa and Binge Eating Disorder Following a 3-Week Inpatient Multidisciplinary Rehabilitation Program

**DOI:** 10.3390/jcm14197127

**Published:** 2025-10-09

**Authors:** Anna Guerrini Usubini, Sara Ducale, Adele Bondesan, Diana Caroli, Francesca Frigerio, Sandra Savino, Laura Abbruzzese, Gianluca Castelnuovo, Alessandro Sartorio

**Affiliations:** 1 Experimental Laboratory for Auxo-Endocrinological Research, Istituto Auxologico Italiano, Istituto di Ricovero e Cura a Carattere Scientifico IRCCS, 28824 Piancavallo-Verbania, Italy; a.bondesan@auxologico.it (A.B.); d.caroli@auxologico.it (D.C.); f.frigerio@auxologico.it (F.F.); sartorio@auxologico.it (A.S.); 2 Psychology Research Laboratory, Istituto Auxologico Italiano, Istituto di Ricovero e Cura a Carattere Scientifico IRCCS, 28824 Piancavallo-Verbania, Italy; s.ducale@auxologico.it (S.D.); gianluca.castelnuovo@auxologico.it (G.C.); 3 Division of Eating and Nutrition Disorders, Istituto Auxologico Italiano, Istituto di Ricovero e Cura a Carattere Scientifico IRCCS, 28824 Piancavallo-Verbania, Italy; s.savino@auxologico.it; 4 Division of Auxology, Istituto Auxologico Italiano, Istituto di Ricovero e Cura a Carattere Scientifico IRCCS, 28824 Piancavallo-Verbania, Italy; l.abbruzzese@auxologico.it; 5 Department of Psychology, Catholic University of Milan, 20123 Milan, Italy

**Keywords:** eating disorders, inpatient rehabilitation, psychological well-being, SDQ, PGWBI, pre–post design

## Abstract

**Background:** This study examined changes in psychological adjustment among patients with Anorexia Nervosa (AN) and Binge Eating Disorder (BED) following a 3-week inpatient multidisciplinary (disease-tailored) rehabilitation program. **Methods**: twenty consecutive Italian female adults with a diagnosis of AN (mean age ± SD: 25.9 ± 9.4 years; mean Body Mass Index: BMI: kg/m^2^: 15.8 ± 1.61) and fifteen consecutive Italian female adults with diagnosis of BED (mean age ± SD: 43.5 ± 15.3 years; mean Body Mass Index: BMI: kg/m^2^: 41.1 ± 7.82) were admitted to the study. Psychological functioning and well-being were assessed pre- and post-intervention using the Strengths and Difficulties Questionnaire and the Psychological General Well-Being Index. **Results**: Significant improvements in emotional symptoms [F(1, 31) = 21.1973, *p* < 0.001, ƞ^2^p = 0.406] and overall psychological functioning [F(1, 31) = 10.0062, *p* = 0.373, ƞ^2^p = 0.026] were observed in both groups, with the most pronounced changes in internalizing symptoms, such as anxiety and depression. Changes in BMI were significantly associated with emotional symptoms, vitality [F(1, 31) = 4.89, *p* = 0.035, ƞ^2^p = 0.136], and total well-being scores [F(1, 31) = 6.341, *p* = 0.017, ƞ^2^p = 0.170]. By contrast, no significant changes were observed in domains such as behavioral problems, hyperactivity/inattention, and peer relationships, probably indicating the need for more prolonged and targeted, domain-specific interventions. **Conclusions**: A 3-week inpatient multidisciplinary program was associated with improvements in internalizing symptoms and psychological well-being in women with AN and BED. Domains such as behavioral regulation and social functioning showed limited change, indicating the need for longer and targeted psychosocial components.

## 1. Introduction

Eating disorders (EDs) are severe and complex psychiatric conditions characterized by an intense focus on body weight and shape and disturbances in eating behaviors, such as restrictions and binge eating [1]. Commonly, EDs first appear in adolescence and are more prevalent in females [2,3,4]. EDs are linked to higher mortality and suicide rates [5]. In addition to these risks, EDs are also associated with severe physical health complications and significant psychological distress [6,7]. Among EDs, Anorexia Nervosa (AN) is characterized primarily by restrictive eating, intense fear of weight gain, and a distorted body image, often leading to a significantly low body weight. In contrast, Binge Eating disorder (BED) involves recurrent episodes of consuming unusually large amounts of food accompanied by a sense of a loss of control, typically without compensatory behaviors such as purging. Despite these differences, both disorders share core maintaining mechanisms described in cognitive behavioral therapy (CBT), including overvaluation of shape and weight, rigid dietary rules, and maladaptive coping strategies [8]. Both groups often experience high levels of distress, low self-esteem, and difficulties in emotional regulation, which can persist even after weight restoration or a reduction in eating disorder symptoms. Addressing psychological adjustment is therefore critical, as improvement in weight or symptom remission alone does not necessarily translate into long-term recovery. Enhancing psychological well-being can contribute to relapse prevention, support more stable functioning in daily life, and improve overall quality of life.

Treatment for EDs is generally provided along a continuum of care, starting with outpatient services and progressing to more intensive levels of intervention, including intensive outpatient programs, day-hospital treatment, or partial hospitalization, residential care, and, ultimately, inpatient hospitalization [9]. The level of care required is determined by the severity of the individual’s medical and psychological condition.

Residential and inpatient treatment programs are generally employed for individuals with severe or treatment-resistant EDs, providing intensive, multidisciplinary care [10]. According to international clinical guidelines, such interventions are particularly recommended when outpatient treatment has proven ineffective or when significant medical or psychiatric risks are present [11].

Despite the widespread use of intensive treatment settings for adults with EDs, there is limited research on the effectiveness and outcomes of these facilities [9]. In particular, while inpatient hospitalization primarily focuses on physical health and maladaptive eating behaviors, its effects on broader psychological adjustment and perceived well-being are less understood. This gap is particularly concerning given the substantially higher costs of these programs compared to outpatient interventions. Furthermore, addressing this gap is crucial to enhancing the effectiveness and efficiency of intensive treatment programs.

The present study aimed to examine changes in psychological adjustment in patients with EDs undergoing 3-week inpatient multidisciplinary treatment. In particular, we compared psychological well-being and adjustment in young adult and adult patients with AN and BED before (pre-intervention) and after (post-intervention) a 3-week in-hospital multidisciplinary rehabilitation program for EDs. Specifically, we hypothesized greater improvements in internalizing symptoms and PGWBI domains compared to behavioral or social domains after 3 weeks.

## 2. Materials and Methods

### 2.1. Participants and Procedures

Participants were recruited at the Division of Eating and Nutrition Disorders, Istituto Auxologico Italiano IRCCS, Piancavallo (VB), a third-level clinical center located in Northern Italy, specialized in the multidisciplinary treatment of severe obesity and EDs. Inclusion criteria were: (1) being an Italian female; (2) being aged between 18 and 70 years; and (3) being diagnosed with AN and BED. The clinical diagnosis was derived from a clinical interview and examination based on the Diagnostic and Statistical Manual of Mental Disorders, 5th Edition (DSM-5) criteria. Participants were excluded if they had any form of severe physical or mental impairment that could compromise their participation in the study.

After being informed about the research and obtaining written informed consent to participate, participants were screened to determine their eligibility for the study. The clinical interview was conducted by an independent psychologist with expertise in clinical psychology to assess inclusion/exclusion criteria.

All patients who were asked to participate met all the inclusion criteria. Recruitment took place during the inpatient admission period, which facilitated complete participation and helped prevent dropouts. All eligible patients agreed to take part, and therefore, no refusals or withdrawals were recorded. Once enrolled, participants self-reported their socio-demographic data and completed two self-report questionnaires, the first one administered at the beginning of a 3-week in-hospital multidisciplinary rehabilitative program (the second day after entering the hospital, i.e., pre-intervention), and the second one at the end of the treatment, just before discharge from the hospital (post-intervention). Outcome assessors were members of the research team and were independent from the care team. Data were collected between November 2023 and April 2024.

The current study was approved by the Ethical Committee of Istituto Auxologico Italiano, IRCCS, Milan, Italy, on 17 May 2022 (EC reference number: 2022_05_17_08; research project code: 03C214). Research was conducted in accordance with the Declaration of Helsinki and its subsequent revisions.

### 2.2. Measures

Socio-demographic data: Participants self-reported information about age, educational level, and socioeconomic status.

Anthropometric data: Weight and height were measured by the internal medical staff to calculate Body Mass Index (BMI: kg/m^2^). Standing height was determined by a Harpenden Stadiometer (Holtain Limited, Crymych, Dyfed, UK). Weight was measured to the nearest 0.1 kg using an electronic scale (RoWU 150, Wunder Sa.bi., Trezzo sull’Adda, Italy).

Psychological adjustment: The Italian-validated version [11] of the Strengths and Difficulties Questionnaire (SDQ) [12] for adults was used. The SDQ, a self-report questionnaire that addresses psychological adjustment, is composed of 25 items rated on a 3-point Likert scale (0 = not true, 1 = somewhat true, 2 = certainly true), assessing the following subscales: Emotional Symptoms, Conduct Problems, Hyperactivity–Inattention, Peer Problems, and Prosocial Behavior. By summing the scores of all the subscales, excluding the “Prosocial Behavior” subscale, the Total Difficulties score is obtained (range score: from 0 to 40 points). The original version of SDQ [13] showed good internal consistency. The internal consistency of the Italian version was good, with Cronbach’s alpha values for the subscales ranging from 0.60 to 0.85. The SDQ and relative information about administration and interpretation are available free of charge and can be accessed online at https://sdqinfo.org/.

Psychological well-being: The Psychological General Well-Being Index (PGWBI; [14]) was used to assess psychological well-being. The PGWBI is a self-report questionnaire designed to assess subjective psychological well-being and distress across six dimensions: anxiety, depression, positive well-being, self-control, general health, and vitality. It comprises 22 items rated on a 6-point Likert scale, with higher scores indicating greater well-being. The Italian version [15], validated for use in clinical and general populations, has demonstrated good psychometric properties, with Cronbach’s alpha values for the subscales ranging from 0.70 to 0.94.

### 2.3. Three-Week In-Hospital Multidisciplinary Rehabilitation Program for EDs

Patients participated in a disease-tailored inpatient multidisciplinary program for EDs. The 3-week rehabilitation program has general goals of improving the patient’s overall physical and psychological well-being and promoting healthier eating behaviors by restoring physiological eating habits. This is achieved through the development of self-management skills in relation to eating behavior and the recognition of the emotional factors that influence it.

In cases where an eating disorder is present, the program also aims to provide tools for recognizing biological hunger and satiety, distinguishing them from emotional hunger and satiety. This helps patients acquire the skills needed to manage compulsive eating episodes, motor hyperactivity, food refusal, and compensatory behaviors. Within a short residential program, improvements are most likely to occur in the behavioral and nutritional domains. Patients may benefit from more regular eating routines, partial weight stabilization, and a reduction in acute risk behaviors such as restrictive fasting, purging, or excessive exercise. On the emotional side, patients can begin to develop greater awareness of their difficulties, gain initial tools for emotional regulation, and experience increased motivation for recovery. During this period, patients with AN were treated with a personalized nutritional rehabilitation program aimed at promoting weight regain (0.5–1.4 kg/week) and refeeding practice, in line with the guidelines [16,17]. Supplementation with vitamins was prescribed when necessary. All the meals were assisted and supervised by the team of dietitians. The patients participated in both individual and group nutritional counseling sessions, coordinated by the team of dieticians, with the goal of promoting behavioral changes. When clinical conditions permitted, the intervention also included a daily, adapted (light) physical activity program (15–30 min), implemented by physiotherapists and certified trainers, to promote muscle mass recovery and improve body composition. With psychotherapists, the patients received individual and group sessions of cognitive behavioral psychotherapy twice a week.

Patients with BED were also placed on a tailored, nutritionally balanced diet. The prescribed energy intake was calculated by subtracting approximately 500 kcal from the estimated basal energy expenditure. The diet was composed of 21% protein, 53% carbohydrates, and 26% lipids. Subsequently, they engaged in both individual and group nutritional counseling sessions, coordinated by the team of nutritionists/dieticians, with the objective of facilitating sustainable changes in dietary behaviors. The intervention also included a daily adapted physical activity program, implemented by physiotherapists and certified trainers, to enhance muscle strength and improve body composition, as well as a therapeutic intervention targeting body image twice a week. The physical exercise program consisted of 5 days per week of training, including (i) 1 h of dynamic aerobic standing and floor exercises with arms and legs (i.e., squats, step ups, jump rope, lunges, push-ups, torso twists), at moderate intensity (monitored through a portable heart rate monitor [Polar RS400SD, Polar Electro Oy, Kempele, Finland]); and (ii) either 20–30 min cycle ergometer exercise at 60 W, or 3–4 km outdoor walking on flat terrain, according to individual capabilities and clinical status. The subjects also underwent a psychological counseling program (i.e., cognitive behavioral therapy strategies, such as stimulus control procedures, problem-solving, and stress management training, development of healthy eating habits, assertiveness and social skills training, cognitive restructuring of negative maladaptive thoughts, and relapse prevention training) consisting of one individual and one group session per week of individual and/or group psychotherapy performed by clinical psychologists. Furthermore, lectures on the problems and risks of obesity, motivational speeches, examples of healthy foods, food preparation workshops, and group discussions, with or without a supervisor, took place daily.

### 2.4. Statistical Analysis

The sample size has been calculated for a priori mixed between–within repeated measures Analysis of Variance (ANOVA) using G*Power software (3.1.9.4). Setting alpha to 0.05, power to 0.80, and correlation among repeated measures to 0.5, a total sample of 34 is considered sufficient to detect a medium effect size (f = 0.25).

Categorical variables were presented as frequencies and percentages, while continuous variables were presented as means and standard deviations. To assess the normal distribution of the variables, the skewness and kurtosis indices were assessed. Statistical analyses were conducted using mixed-design repeated measures analyses of variance (ANOVAs) to examine changes from pre- to post-intervention in patients with AN and those with BED in psychological strengths and difficulties (SDQ) and psychological well-being (PGWBI). Time (pre-intervention vs. post-intervention) was treated as the within-subjects factor, and diagnostic group (AN vs. BED) as the between-subjects factor. Separate ANOVAs were performed for each subscale of the SDQ and PGWBI. For each analysis, the main effects of Time and Group, as well as the Time × Group interaction, were evaluated. To control for the potential influence of weight changes on psychological outcomes, change in BMI (ΔBMI = BMI post-intervention − BMI pre-intervention) was included as a covariate. Assumptions of normality, and homogeneity of variances were assessed prior to analysis. The assumption of sphericity is automatically satisfied, as there is only one possible difference between the two measurements. Therefore, no corrections for violations of sphericity, such as the Greenhouse–Geisser correction, were required. Similarly, no post hoc tests or Bonferroni correction were required, as there is only a single possible comparison. Effect sizes were reported using partial eta squared (η^2^ₚ), and statistical significance was set at *p* < 0.05. All analyses were performed using Jamovi (2.6.26).

## 3. Results

The sample was composed of twenty consecutive Italian female adults with a diagnosis of Anorexia Nervosa (AN) (mean age ± SD: 25.9 ± 9.4 years; mean Body Mass Index: BMI: kg/m^2^: 15.8 ± 1.61) and fifteen consecutive Italian female adults with diagnosis of Binge Eating Disorder (BED) (mean age ± SD: 43.5 ± 15.3 years; mean Body Mass Index: BMI: kg/m^2^: 41.1 ± 7.82). In the AN group, 15 (75%) participants lived in the North of Italy, 11 (55%) had a high school degree, and 15 (75%) were students. In the BED group, 14 (93.3%) lived in the North of Italy, 9 (60%) completed lower secondary school, and 8 (53.3%) were unemployed. The prevalence of individuals from Northern Italy in the sample was probably dependent on the location of the Institute in the same regions. Descriptive analyses conducted prior to the intervention revealed significant demographic and clinical differences between the two groups. Participants with AN had a significantly lower mean age (Welch’s t (21.8) = 3.93; *p* < 0.001; Cohen’s d = 1.38; 95% CI = 0.673–2.18) and, obviously, a lower body weight and BMI (Welch’s t (14.9) = 12.29; *p* < 0.001; Cohen’s d = 4.47; 95% CI = 3.468–6.14) than those participants diagnosed with BED. From pre- to post-intervention, BMI in the AN group significantly increased (*p* = 0.021). Conversely, in the BED group, BMI significantly decreased from pre- to post-intervention (*p* < 0.001).

Results are presented in Table 1.

### 3.1. Psychological Strengths and Difficulties

#### 3.1.1. Qualitative Interpretation of SDQ

Each subscale and total SDQ score can be qualitatively interpreted as “normal”, “abnormal” or “borderline” according to the original categorization.

Assuming this qualitative point of view, at pre-intervention, 12 out of 20 participants (60%) in the AN group reported scores on the Emotional Symptoms subscale of the SDQ that fall within the “abnormal” range. In contrast, for the Conduct Problems (60%) and Prosocial Behavior (100%) subscales, the majority of participants scored within the range considered “normal”. Scores classified as “borderline” on the Peer Problems subscale were observed in 7 out of 20 participants (35%), while “abnormal” scores on the Total Difficulties scale were reported in 8 out of 20 participants (40%).

In the post-intervention, the majority of participants scored within the range considered “normal” for all subscales and the Total SDQ score.

In the BED group, most participants fell within the range of “normal score” for all the subscales of SDQ, except for the Emotional Symptoms subscale, in which 47% of participants reported “abnormal” scores. In the post-intervention, the majority of participants scored within the range considered “normal” for all subscales and the Total score of the SDQ, except for the Peer Problems subscale, where “normal scores” were obtained by only 5 out of 15 participants (33%).

Frequencies and percentages of normal, borderline, and abnormal scores of SDQ of the total sample are presented in Table 2.

#### 3.1.2. Quantitative Interpretation of SDQ

To examine changes from pre- to post-intervention in patients with AN and those with BED in terms of psychological strengths and difficulties, different ANOVAs were performed for all subscales and the total score of SDQ. Results revealed significant reductions in Emotional symptoms, Behavioral Problems, and Total score of SDQ, following the intervention across participants, as resulting from a significant main effect of Time in Emotional Symptoms [F(1, 31) = 21.1973, *p* < 0.001, ƞ^2^p = 0.406], and Total score of SDQ [F(1, 31) = 10.0062, *p* = 0.003, ƞ^2^p = 0.244].

No significant effect of Time in Behavioral Problems [F(1, 31) = 3.304, *p* = 0.079, ƞ^2^p = 0.096], Hyperactivity/Inattention [F(1, 31) = 3.293, *p* = 0.079, ƞ^2^p = 0.096], Peer Relationships [F(1, 31) = 0.2521, *p* = 0.619, ƞ^2^p = 0.008], and Prosocial Behaviors [F(1, 31) = 0.17247, *p* = 0.681, ƞ^2^p = 0.006] was found.

No significant effect of Diagnosis in Emotional Symptoms [F(1, 31) = 0.188, *p* = 0.095, ƞ^2^p = 0.006], Behavioral problems [F(1, 31) = 0.188, *p* = 0.668, ƞ^2^p = 0.006], Hyperactivity/Inattention [F(1, 31) = 0.0165, *p* = 0.899, ƞ^2^p = 0.001], Peer relationships [F(1, 31) = 0.2467, *p* = 0.623, ƞ^2^p = 0.008], Prosocial Behaviors [F(1, 31) = 0.861, *p* = 0.361, ƞ^2^p = 0.027] and Total score of SDQ [F(1, 31) = 0.816, *p* = 0.373, ƞ^2^p = 0.026] were found, indicating that there were no significant differences between AN and BED on these measures, irrespective of time.

No significant Time × Diagnosis interaction was found, suggesting that the pattern of change over time did not differ significantly between the two diagnostic groups.

Changes in body weight over time were accounted for in the analyses. Specifically, variation in BMI (ΔBMI) was included as a covariate to control for the potential impact of weight change on psychological outcomes. In particular, in the AN group, mean±SD BMI changed from pre- to post-intervention from 15.8 ± 1.61 to 16.3 ± 1.60 (mean±SD ΔBMI: 0.529 ± 0.909 min-max: −1.25–2.10). In the BED group, mean±SD BMI changed from pre- to post-intervention from 41.1 ± 7.86 to 39.5 ± 7.56 (mean±SD ΔBMI: −1.58 ± 0.965 min-max: −3.38–0.175). ΔBMI had a significant main effect on Emotional Symptoms [F(1, 31) = 10.55, *p* = 0.003, ƞ^2^p = 0.254], and a marginally significant effect on Total score of SDQ [F(1, 31) = 3.892, *p* = 0.057, ƞ^2^p = 0.112], suggesting that participants with greater changes in BMI, regardless of whether these changes constituted an increase or decrease, showed different overall levels of psychological adjustment.

No other significant main effect of ΔBMI was found.

### 3.2. Psychological Well-Being

As far as psychological well-being is concerned, ANOVAs regarding changes from pre- to post-intervention in patients with AN and those with BED in psychological well-being, revealed a significant improvement in Anxiety, Depression, Positive Well-Being, Self-Control, General Health and Total score of PGWBI, as resulting from the main effect of Time in Anxiety [F(1, 31) = 15.15, *p* < 0.001, ƞ^2^p = 0.328], Depression [F(1, 31) = 11.52, *p* = 0.002, ƞ^2^p = 0.271], Positive Well-Being [F(1, 31) = 17.780, *p* < 0.001, ƞ^2^p = 0.364], Self-Control [F(1, 31) = 13.697, *p* < 0.001, ƞ^2^p = 0.306], General Health [F(1, 31) = 12.515, *p* < 0.001, ƞ^2^p = 0.288], Vitality [F(1, 31) = 28.370, *p* < 0.001, ƞ^2^p = 0.478], and Total score of PGWBI [F(1, 31) = 15.044, *p* < 0.001, ƞ^2^p = 0.327].

No significant effect of Diagnosis of Anxiety [F(1, 31) = 0.0125, *p* = 0.912, ƞ^2^p = 0.000], Depression [F(1, 31) = 1.31, *p* = 0.262, ƞ^2^p = 0.040], Positive Well-Being [F(1, 31) = 0.125, *p* = 0.726, ƞ^2^p = 0.004], Self-Control [F(1, 31) = 1.25 × 10^−6^, *p* = 0.999, ƞ^2^p = 0.000], General Health [F(1, 31) = 1.39, *p* = 0.247, ƞ^2^p = 0.043], Vitality [F(1, 31) = 2.15, *p* = 0.153, ƞ^2^p = 0.065], and Total score of PGWBI [F(1, 31) = 0.493, *p* = 0.488, ƞ^2^p = 0.016], were found, indicating that there were no significant differences between AN and BED on these measures, irrespective of time.

No significant Time x Diagnosis interactions were found, suggesting that the pattern of change in other measures over time did not differ significantly between the two diagnostic groups.

ΔBMI had a significant main effect in Vitality [F(1, 31) = 4.89, *p* = 0.035, ƞ^2^p = 0.136], and Total score of PGWBI [F(1, 31) = 6.341, *p* = 0.017, ƞ^2^p = 0.170]. Specifically, participants who experienced larger changes in BMI, regardless of whether such changes constituted an increase or a decrease, tended to report different overall levels of well-being compared to those with smaller BMI changes.

No other significant main effect of ΔBMI was found.

Δ scores (post-pre) for all the subscales of SDQ and PGWBI for the two subgroups were presented in Table 3.

## 4. Discussion

The present study investigated changes in psychological adjustment among patients with AN and BED undergoing a 3-week inpatient multidisciplinary rehabilitation program for EDs.

Overall, the findings indicated a general improvement in psychological well-being and adjustment from pre- to post-intervention, as evidenced by both a descriptive analysis of SDQ scores and quantitative analyses of SDQ and PGWBI outcomes.

From a qualitative standpoint, a substantial proportion of individuals in the AN group presented with “abnormal” scores on the SDQ Emotional Symptoms (60%) and Total Difficulties (40%) subscales at baseline, underscoring the high prevalence of internalizing symptoms—such as anxiety and depression—commonly reported in this population [18,19,20]. Following the intervention, the majority of AN participants scored within the “normal” range across all SDQ subscales, suggesting broad improvements in emotional functioning and interpersonal adjustment.

In the BED group, although most participants exhibited “normal” scores across most SDQ domains at baseline, 46% showed “abnormal” scores on the Emotional Symptoms subscale, reflecting the presence of notable affective symptoms despite relatively intact psychosocial functioning. Post-intervention results revealed normalization across all SDQ domains for the majority of participants. The amelioration of the psychological conditions of patients with BED following the rehabilitation program can be seen in light of existing evidence that supports the effectiveness of structured, intensive inpatient programs in reducing psychological distress and enhancing psychosocial functioning in individuals with EDs [21].

To quantitatively assess changes in psychological adjustment, SDQ scores were analyzed from pre- to post-intervention. The results indicated significant reductions in emotional and behavioral distress across both diagnostic groups. Notably, Emotional Symptoms showed the most pronounced improvement (*p* < 0.001), highlighting a reduction in internalizing difficulties such as anxiety and depression. Similarly, a significant reduction in the Total Difficulties score of SDQ (*p* = 0.003) reflected a more general enhancement in overall psychological functioning. Such improvements in internalizing symptoms may plausibly stem from factors such as nutritional and emotional stabilization [22], the structured and supportive environment, and the intensive therapeutic input provided during the program [23]. However, further research will be needed to test these hypotheses empirically.

In contrast, no significant changes were observed over time in Behavioral Problems (*p* = 0.079), Hyperactivity/Inattention (*p* = 0.079), Peer Relationship Problems (*p* = 0.619), and Prosocial Behavior (*p* = 0.681). These findings suggest that such domains may be less responsive to the intervention provided, possibly due to the relatively short treatment period, or may require more targeted, domain-specific approaches to produce meaningful change.

Importantly, no significant main effects of diagnosis were found for any SDQ subscales or the Total Difficulties score, indicating that overall levels of psychological adjustment did not significantly differ between diagnostic groups. Likewise, the absence of a significant Time × Diagnosis interaction suggests that the trajectory of change from pre- to post-intervention was comparable between patients with AN and those with BED, indicating a similar psychological response to treatment, regardless of diagnosis.

However, a significant main effect of ΔBMI (change in BMI over the time of intervention) was found for Emotional Symptoms, and a marginally significant effect was observed for the Total Difficulties score. The findings indicate that changes in BMI were significantly associated with emotional functioning. The partial eta-squared suggests a large effect size, meaning that ΔBMI accounted for a substantial proportion of the variance in emotional symptoms. Additionally, ΔBMI showed a marginally significant effect on the Total SDQ score, indicating a potential, though less robust, impact on overall psychological adjustment.

These results suggest that participants experiencing larger changes in BMI—regardless of whether it was an increase or decrease—tended to exhibit differences in emotional well-being and, to a lesser extent, overall psychological adjustment. No other domains of the SDQ were significantly affected, highlighting that ΔBMI may be particularly relevant for emotional aspects of psychological functioning in the context of the rehabilitation program.

Regarding well-being, both diagnostic groups exhibited substantial improvements across all PGWBI subscales from pre- to post-intervention, further supporting the beneficial impact of the inpatient program on psychological adjustment and overall well-being [24,25,26].

Consistent with findings from the SDQ, no significant main effect of diagnosis was observed for any PGWBI dimension, indicating that patients with AN and BED experienced comparable levels of psychological well-being.

Additionally, ΔBMI was significantly associated with outcomes on the Vitality subscale (*p* = 0.035) and the PGWBI Total score (*p* = 0.017), suggesting that greater changes in BMI, regardless of whether these changes constituted an increase or decrease, were linked to variations in perceived psychological well-being. In other words, differences in BMI appear to be a significant factor in well-being, highlighting the interplay between physical and psychological outcomes in multidisciplinary interventions for weight management or eating disorder rehabilitation.

The findings of the present study should be interpreted in light of several limitations. The uncontrolled pre-post design of the study, the small sample size of Italian females (predominantly from Northern Italy), and the lack of a control group limit generalizability and preclude causal inferences regarding the effectiveness of the rehabilitation intervention in improving the psychological conditions of patients. In addition, the small sample size prevented us from including additional covariates (e.g., age and education) in the models. While such adjustments could help address potential group imbalances, the inclusion of several covariates with a limited number of participants would substantially reduce statistical power and increase the likelihood of overfitting.

To address these limitations, future replications of the study should include a control group of normal-weight individuals, a larger sample size with a more representative sample of the Italian population, and follow-up measures to verify whether psychological improvements are maintained after discharge. This approach would enable a more rigorous assessment of whether the improvements in psychological well-being are directly attributable to the intervention, rather than to extraneous factors. Moreover, the homogeneity of the sample (e.g., all females, predominantly from Northern Italy) may not reflect the broader population of individuals with EDs. While women continue to represent the majority of cases, emerging evidence suggests that men are increasingly affected by various forms of EDs [27]. Future research should aim to replicate these findings in more varied and larger samples and explore the sustainability of improvements over time through follow-up assessments. In addition, both the SDQ and PGWBI are self-report instruments, which may be subject to response biases, such as social desirability.

Despite the above limitations, this study provides valuable insights into the psychological changes experienced by individuals with EDs following participation in an inpatient multidisciplinary rehabilitation program. By including both individuals with AN and BED, the study offers comparative data that may contribute to the development of more tailored and diagnosis-specific interventions. Notably, the findings indicated that while overall psychological well-being and internalizing symptoms—such as anxiety and depression—tend to show significant improvement during the treatment, other domains, including behavioral regulation, attention, and social functioning, appear to be less responsive to change. These different outcomes suggest that a standardized treatment approach may not be sufficient for addressing the full spectrum of psychological difficulties associated with EDs. Instead, they emphasize the importance of incorporating more individualized and long-term therapeutic strategies, potentially integrating targeted psychological and psychosocial components aimed at enhancing functioning in these specific areas.

Future studies should consider incorporating randomized or comparator group designs, extending follow-up periods to assess long-term impact, developing targeted modules tailored to specific subgroups, and expanding inclusion criteria to include men to enhance generalizability.

## 5. Conclusions

A 3-week inpatient multidisciplinary program was associated with improvements in internalizing symptoms and psychological well-being in women with AN and BED. Domains such as behavioral regulation and social functioning showed limited change, indicating a need for longer and more targeted psychosocial interventions.

## Figures and Tables

**Table 1:** Means and standard deviations of the clinical variables at pre-intervention and post-intervention.

		Pre- Intervention		Post- Intervention	
		AN Group M (SD)	BED Group M (SD)	AN Group M (SD)	BED Group M (SD)
SDQ	Emotional Symptoms	6.80 (2.67)	6.00 (2.51)	5.15 (2.62)	4.47 (2.64)
	Conduct Problems	3.00 (2.08)	2.87 (2.47)	2.75 (1.97)	1.93 (2.34)
	Hyperactivity/Inattention	5.45 (2.72)	3.60 (2.41)	4.85 (2.70)	3.00 (2.48)
	Peer Problems	3.30 (2.05)	4.07 (2.96)	3.05 (1.99)	4.00 (2.80)
	Prosocial Behaviors	8.15 (1.46)	7.87 (1.36)	8.15 (1.79)	8.20 (1.26)
	Total Difficulties	18.55 (7.98)	16.33 (7.83)	15.80 (7.60)	13.40 (8.09)
PGWBI	Anxiety	8.85 (6.38)	12.00 (5.73)	12.35 (6.39)	16.67 (6.60)
	Depression	4.70 (4.04)	8.87 (3.98)	11.85 (11.60)	10.13 (4.37)
	Positive well-being	4.10 (3.67)	5.93 (2.34)	9.05 (4.16)	8.27 (4.92)
	Self-control	6.00 (2.71)	8.33 (4.13)	10.35 (5.41)	9.13 (4.14)
	General health	6.25 (2.81)	7.00 (3.46)	9.90 (4.24)	8.27 (2.76)
	Vitality	5.65 (4.13)	7.20 (3.34)	11.25 (3.51)	10.00 (5.98)
	Total score	35.55 (19.05)	49.33 (17.68)	56.45 (19.07)	62.47 (23.94)

Note: SDQ: Strengths and Difficulties Questionnaire; PGWBI: Psychological General Well-Being Index. Δ score: (post-pre).

**Table 2:** Frequencies and percentages of abnormal, borderline, and normal scores of SDQ.

		Pre-Intervention		Post-Intervention	
		AN Group	BED Group	AN Group	BED Group
SDQ					
	Emotional Symptoms	Abnormal: 12 (60%)	Abnormal: 7 (47%)	Abnormal: 7 (35%)	Abnormal: 4 (27%)
		Borderline: 1 (5%)	Borderline: 1 (5%)	Borderline: 2 (10%)	Borderline: 1 (7%)
		Normal: 7 (35%)	Normal: 7 (47%)	Normal: 11 (55%)	Normal: 10 (66%)
	Conduct Problems	Abnormal: 5 25(%)	Abnormal: 3 (20%)	Abnormal: 4 (20%)	Abnormal: 2 (13%)
		Borderline: 3 (15%)	Borderline: 2 (13%)	Borderline: 3 (15%)	Borderline: 0
		Normal: 12 (60%)	Normal: 10 (67%)	Normal: 13 (65%)	Normal: 13 (87%)
	Hyperactivity/Inattention	Abnormal: 9 (45%)	Abnormal: 1 (7%)	Abnormal: 6 (30%)	Abnormal: 0
		Borderline: 1 (5%)	Borderline: 2 (13%)	Borderline: 3 (15%)	Borderline: 0
		Normal: 10 (50%)	Normal: 12 (80%)	Normal: 11 (55%)	Normal: 15 (100%)
	Peer Problems	Abnormal: 3 (15%)	Abnormal: 0	Abnormal: 2 (10%)	Abnormal: 5 (33%)
		Borderline: 7 (35%)	Borderline: 6 (40%)	Borderline: 6 (30%)	Borderline: 5 (33%)
		Normal: 10 (50%)	Normal: 9 (60%)	Normal: 12 (60%)	Normal:5 (33%)
	Prosocial Behaviors	Abnormal: 3 (15%)	Abnormal: 0	Abnormal: 2 (10%)	Abnormal: 5 (33%)
		Borderline: 0	Borderline: 0	Borderline: 0	Borderline: 0
		Normal: 20 (100%)	Normal: 15 (100%)	Normal: 20 (100%)	Normal: 15 (100%)
	Total Difficulties	Abnormal: 8 (40%)	Abnormal: 6 (40%)	Abnormal: 7 (35%)	Abnormal: 3 (20%)
		Borderline: 6 (30%)	Borderline: 1 (7%)	Borderline: 2 (10%)	Borderline: 4 (27%)
		Normal: 6 (30%)	Normal: 8 (53%)	Normal: 11 (55%)	Normal:8 (53%)

Note: SDQ: Strengths and Difficulties Questionnaire.

**Table 3:** Δ scores of SDQ and PGWBI in the AN and BED groups.

		AN Group (N = 20)			BED Group (N = 15)		
		Δ Scores (Post-Pre)	Cohen’s d Effect Size	95% CI	Δ Scores (Post-Pre)	Cohen’s d Effect Size	95% CI
SDQ							
	Emotional Symptoms	−1.65	−0.92	−1.44–−0.39	−1.53	−0.91	−1.50–−0.29
	Conduct Problems	−0.25	−0.23	−0.67–0.21	−0.93	−0.56	−1.10–−0.01
	Hyperactivity/Inattention	−0.60	−0.40	−0.86–0.05	−0.60	−0.21	−0.71–0.31
	Peer Problems	−0.25	−0.14	−0.57–0.30	0.14	0.04	−0.48–0.57
	Prosocial Behaviors	0.00	0.00	−0.44–0.44	0.33	0.37	−0.16–0.89
	Total Difficulties	−2.75	−0.70	−1.18–−0.20	−2.93	−0.62	−1.17–−0.06
PGWBI							
	Anxiety	3.50	0.579	0.10–1.05	4.67	0.69	0.11–1.24
	Depression	7.15	0.639	0.15–1.11	1.27	0.32	−0.20–0.83
	Positive well-being	4.95	1.139	0.56–1.70	2.33	0.52	−0.03–1.05
	Self-control	4.35	0.856	0.33–1.36	0.80	0.24	−0.28–0.75
	General health	3.65	0.875	0.35–1.38	1.27	0.30	−0.22–0.82
	Vitality	5.60	1.431	0.79–2.05	2.80	0.49	−0.05–1.02
	Total score	20.90	1.014	0.46–1.55	13.13	0.60	0.04–1.14

Note: SDQ: Strengths and Difficulties Questionnaire; PGWBI: Psychological General Well-Being.

## Data Availability

Raw data will be uploaded to www.zenodo.org immediately after the manuscript’s acceptance, and it will be made available upon reasonable request to the authors, A.G.U. and A.S.
